# Differential expression of upstream stimulatory factor (USF) 2 variants in eutopic endometria from women with endometriosis: estradiol regulation

**DOI:** 10.1186/s40659-015-0047-2

**Published:** 2015-10-09

**Authors:** Jazmin Castro, Germán Araya, Pamela Inostroza, Paulina Hidalgo, Reinaldo González-Ramos, Hugo Sovino, M. Angélica Boric, Ariel Fuentes, M. Cecilia Johnson

**Affiliations:** Faculty of Medicine, Institute of Maternal and Child Research, University of Chile, P.O. Box 226-3, Santiago, Chile; San Borja-Arriarán Clinical Hospital, Santiago, Chile

**Keywords:** USF2, GPER1, Endometriosis, Eutopic endometrium, Estrogen receptor, Estrogen receptor specific agonists, SF-1, P_450_Arom

## Abstract

**Background:**

Endometriosis, pro-inflammatory and invasive benign disease estrogen dependent, abnormally express in endometria the enzyme P_450_Arom, positively regulated by steroid factor-1 (SF-1). Our objective was to study the nuclear protein contents of upstream stimulating factor 2 (USF2a and USF2b), a positive regulator of SF-1, throughout the menstrual cycle in eutopic endometria from women with and without (control) endometriosis and the involvement of nuclear estrogen receptors (ER) and G-coupled protein estrogen receptor (GPER)-1.

**Results:**

Upstream stimulating factor 2 protein contents were higher in mid (USF2b) and late (USF2a and USF2b) secretory phase in eutopic endometria from endometriosis than control (p < 0.05). In isolated control epithelial cells incubated with E_2_ and PGE_2_, to resemble the endometriosis condition, the data showed: (a) significant increase of USF2a and USF2b nuclear protein contents when treated with E_2_, PPT (specific agonist for ERα) or G1 (specific agonist for GPER1); (b) no increase in USF2 binding to SF-1 E-Box/DNA consensus sequence in E_2_-treated cells; (c) USF2 variants protein contents were not modified by PGE_2_; (d) SF-1 nuclear protein content was significantly higher than basal when treated with PGE_2_, E_2_ or G1, stimulation unaffected by ICI (nuclear ER antagonist); and (e) increased (p < 0.05) cytosolic protein contents of P_450_Arom when treated with PGE_2_, E_2_, PPT or G1 compared to basal, effect that was additive with E_2_ + PGE_2_ together. Nevertheless, in endometriosis cells, the high USF2, SF-1 and P_450_Arom protein contents in basal condition were unmodified.

**Conclusion:**

These data strongly suggest that USF2 variants and P_450_Arom are regulated by E_2_ through ERα and GPER1, whereas SF-1 through GPER1, visualized by the response of the cells obtained from control endometria, being unaffected the endogenously stimulated cells from endometriosis origin. The lack of E_2_ stimulation on USF2/SF-1 E-Box/DNA-sequence binding and the absence of PGE_2_ effect on USF2 variants opposite to the strong induction that they exert on SF1 and P450 proteins suggest different mechanisms and indirect regulations. The sustained USF2 variants protein expression during the secretory phase in eutopic endometria from women with endometriosis may participate in the pathophysiology of this disease strongly associated with infertility and its characteristic endometrial invasion to ectopic sites in the pelvic cavity.

## Background

Endometriosis is an estrogen-dependent gynecologic disease, characterized by the presence and growth of endometrium outside the uterine cavity. This pathology affects about 10 % of reproductive-age women and is associated with infertility, chronic pelvic pain, dysmenorrhea, and dyspareunia [1–4]. The etiology of this disease remains incompletely understood [5]. Retrograde menstruation with viable endometrial fragments has been advocated as one of the mechanisms by which the endometrium reaches the peritoneal cavity [6]. However, this theory fails to explain why only a select group of women experiencing retrograde menstruation develops endometriosis [7].

Endometrial estrogenic microenvironment has been shown to be an important factor in the pathophysiology of endometriosis by abnormal expression of enzymes involved in estrogen synthesis and degradation [8, 9]. The activation of CYP19A1 gene induces P_450_Arom expression, the rate-limiting enzyme in conversion of androgens to estrogens. Normally, steroid factor-1 (SF-1) positively regulates the CYP19A1 gene in the ovary, though not in the normal endometrium. Nevertheless, SF-1, expressed in eutopic and ectopic endometria from women with endometriosis, aberrantly activates CYP19A1 and the expression of P_450_Arom in stroma [10–12] or gland [13, 14] in these tissues as has been extensively described favoring this estrogenic microenvironment in this disease.

SF-1 gene is recognized in a region called E-box by upstream stimulatory factor (USF), the ubiquitous transcription factor involved on embryonic development, fertility, stress, growth and lipid and carbohydrate metabolisms [15, 16]. Although two types of USF, USF1 and USF2, have been reported, it is USF2 that shows the highest binding activity on SF-1 promoter and its knockdown results in down-regulation of SF-1 and also of its target gene CYP19A1 in ectopic endometrium from endometriosis women [17]. Two variants of USF2, produced by alternative splicing, have been reported, the bigger USF2a (44 kDa) and the smaller USF2b (38 kDa) by the loss of 67 internal amino acid in the N-terminal domain [15, 18].

Estradiol (E_2_) acts through the classic nuclear estrogen receptors (ER), ERα and ERβ, both strictly regulated by the ovarian steroid hormones during the menstrual cycle, with a predominance of ERα over ERβ in the normal endometrium and reducing their expression during the secretory phase [19–22]. In eutopic endometrium from women with endometriosis, although each ER isoforms are increased, the ERα/ERβ ratio is decreased affecting the normal actions of E_2_ in this tissue [23, 24]. In addition, a membrane receptor called G-protein estrogen receptor 1 (GPER1) presents high affinity for E_2_ in vitro [25, 26] and has been described as potentially responsible of early and non genomic responses of estrogen in several cell lines and tissues including the endometrium [27–29].

The aberrant expression of CYP19A1 and SF-1 genes in eutopic and ectopic endometria of women with endometriosis led us to study USF2 variants protein contents in human endometrium throughout the menstrual cycle and the effect of estrogenic and proinflammatory environments in epithelial cells of eutopic endometrium from women with and without endometriosis. The involvement of nuclear ERα, ERβ or GPER1 in the E_2_ action on USF2 variants, SF-1 and P450Arom protein expression was also evaluated.

## Results

### USF2 protein contents in endometrium throughout the menstrual cycle

**Fig. 1:**
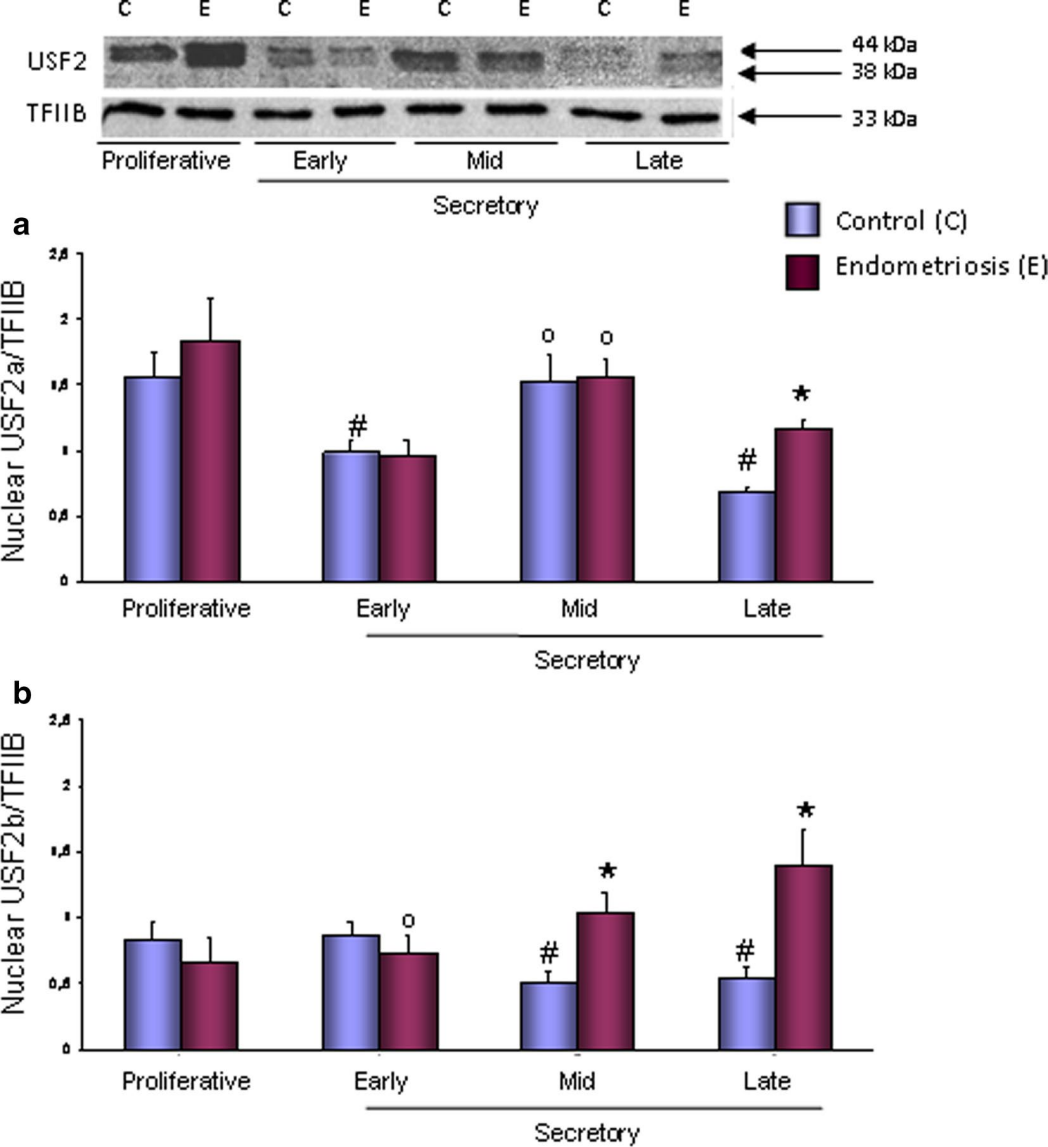
USF2a (**a**) and USF2b (**b**) nuclear protein contents in eutopic endometria throughout the menstrual cycle. Endometria were obtained from seven women without (control) and seven women with endometriosis in each stage of the menstrual cycle. Representative immunoblots are shown. Data were normalized with TFIIB protein contents. Results are the mean ± SEM. *p < 0.05 vs. control; ^#^p < 0.05 vs. proliferative phase; ºp < 0.05 vs. late secretory phase

The protein content of both USF2a and USF2b variants were detected by immunoblot (Fig. 1) in endometrium from women with endometriosis and controls. No interactions between USF2 protein studies and subject age was found by ANCOVA.

Two protein bands (44 and 38 kDa), corresponding to USF2a and USF2b variants, respectively, were found in the nuclear compartment of control and endometriosis endometria (Fig. 1). In control endometria, nuclear USF2a protein content decreased in early and late secretory phases (37 and 57 %, respectively, p < 0.05) as compared to the proliferative phase. On the other hand, in endometriosis, USF2a contents were lower during the late secretory as compared to the mid secretory phase, although higher than late control endometria (Fig. 1a).

During mid and late secretory phases, USF2b (38 kDa) nuclear protein content significantly decreased (39 and 34 %, respectively) as compared to proliferative phase in control endometria, instead it was observed an increase in endometriosis endometria during the same stages of the menstrual cycle, being higher 100 and 155 %, respectively, than control (Fig. 1b).

### Stimulatory effect of E_2_ and PGE2 on USF2, SF-1 and P_450_Arom protein contents

**Fig. 2:**
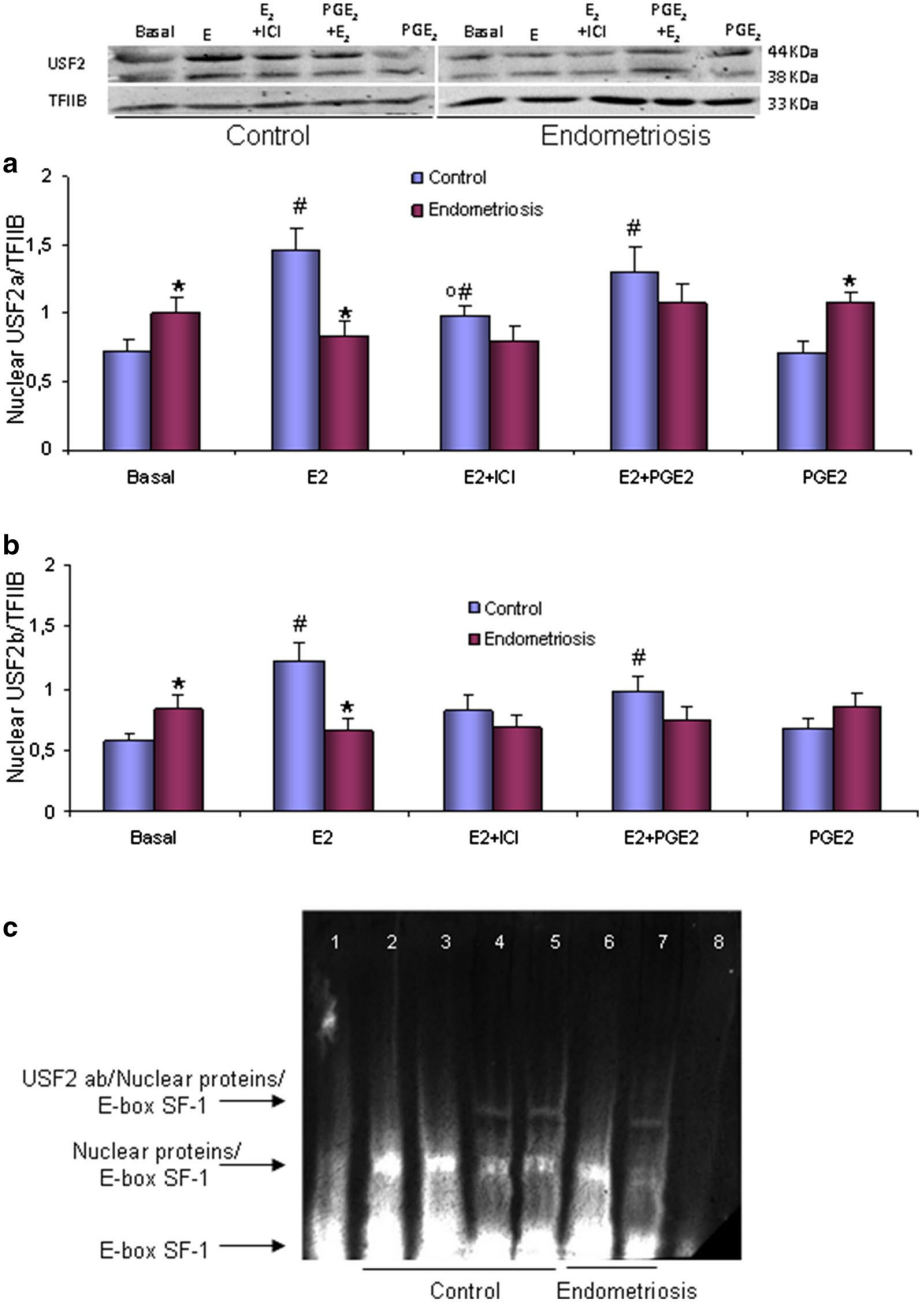
E_2_ and PGE_2_ effect on USF2 variants protein contents and SF-1 E-Box DNA binding. USF2a (**a**) and USF2b (**b**) nuclear protein contents of endometrial epithelial cells (EEC) from women with and without (control) endometriosis treated with E_2_ (10^−8^ mol/L) and/or PGE_2_ (10^−8^ mol/L) in the presence or absence of ICI (10^−6^ mol/L; 30 min previously added); all data were normalized with TFIIB. Representative immunoblot is shown. Results are the mean ± SEM of EEC obtained from 7 control women and 7 women with endometriosis. **c** Representative SF-1 E-Box DNA shift assay from 3 gels. *Lane 1* free probe; *lanes 2*–*5* nuclear protein from control EEC (*2* basal condition; *3* E_2_ treated; *4* basal + anti USF2 antibody; *5* E_2_ + anti USF2 antibody); *lanes 6* and *7*: nuclear protein from endometriosis endometrial epithelial cells (*6* basal condition; *7* basal + anti USF2 antibody), and *lane* *8* basal condition + cold competitor. Protein procurements and assays are described in “Methods”. *p < 0.05 control; ^#^p < 0.05 vs. baseline; ºp < 0.05 vs. E_2_

In endometriosis epithelial cells, nuclear protein contents of USF2a and USF2b were significantly higher in basal condition than control cells. Nevertheless, only in control cells, E_2_ increased USF2a (103 %) and USF2b (91 %) nuclear protein contents, effect also partially blocked by the presence of ICI (Fig. 2a, b). Nuclear protein homogenate obtained from control and endometriosis epithelial cells bound to target E-Box motif, complexes displaced by cold probe. The previous incubation of the nuclear protein homogenates with USF2 antibody shifted partially the protein/E-Box complex in basal or E2-treated conditions (Fig. 2c).

We observed a null effect on USF2a and USF2b nuclear protein contents of 10^−8^ mol/L PGE_2_, in the presence or absence of 10^−8^ mol/L E_2_ in isolated epithelial cells from both control and endometriosis endometria (Fig. 2a, b).

**Fig. 3:**
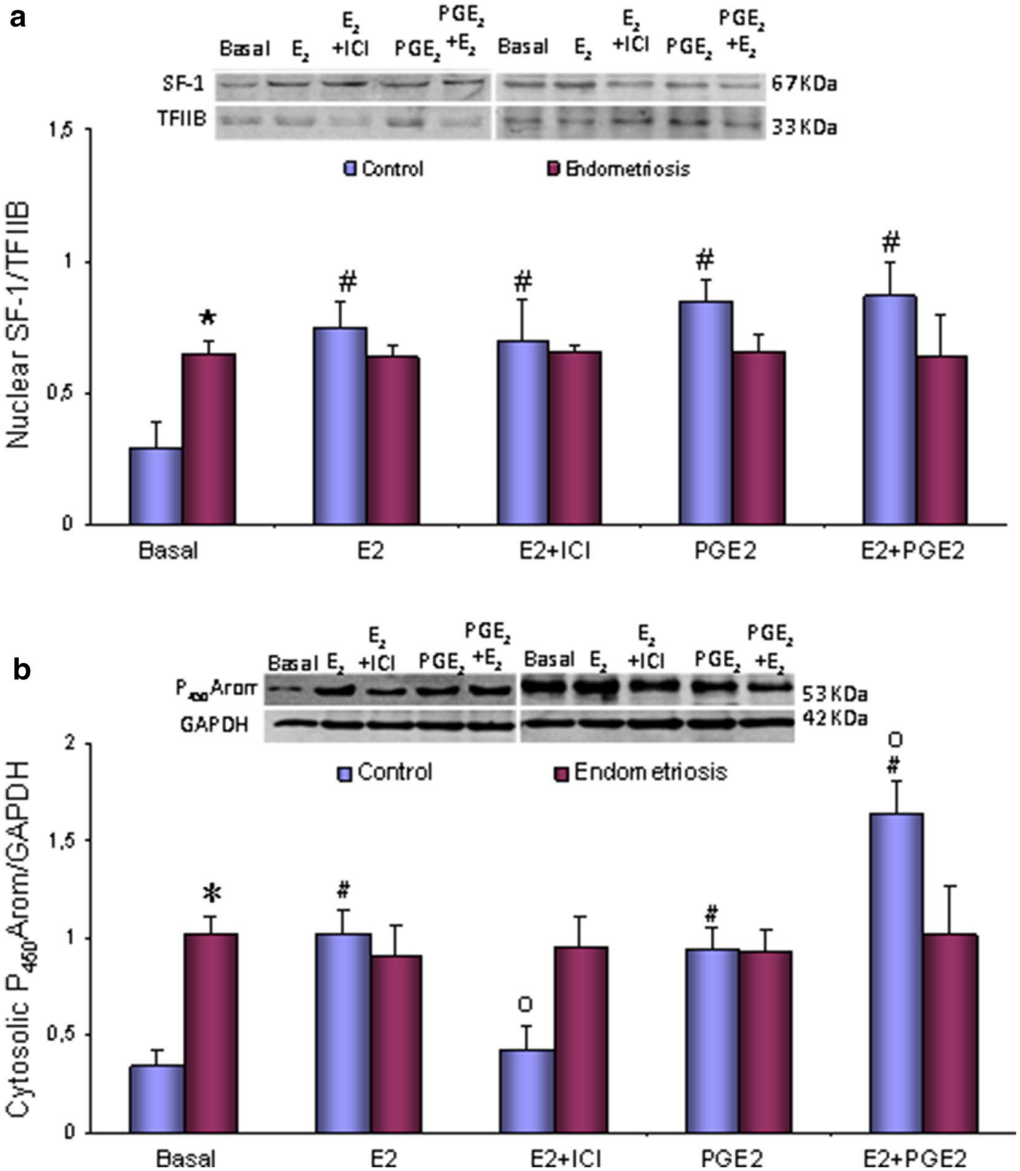
E_2_ and PGE_2_ effect on SF-1 and P_450_Arom protein levels in endometrial epithelial cells. Isolated endometrial epithelial cells (EEC) obtained from 4 to 6 women with or without (control) endometriosis were treated for 24 h with E_2_ (10^−8^ mol/L) and/or PGE_2_ (10^−8^ mol/L) in the presence and absence of ICI (10^−6^ mol/L; 30 min previously added). Representative immunoblots are shown. Data for SF-1 (nuclear homogenates) were normalized with TFIIB (**a**) and for P450Arom (cytosol homogenates) with GAPDH (**b**). Results are the mean ± SEM. *p < 0.05 vs.control; ^#^p < 0.05 vs. basal, ºp < 0.05 vs. one treatment

Epithelial cells obtained from endometriosis endometria had high SF-1 protein expression in basal condition, which were resistant to E_2_ and PGE_2_ (Fig. 3a). On the contrary, in control epithelial cells, the SF-1 protein content was strongly increased by E_2_ (126 %) as compared to basal, effect not modified by the presence of ICI. The presence of PGE_2_ also increased the content of SF-1 protein (154 %) as compared to basal, although no additive or synergistic effects were observed when E_2_ and PGE_2_ were added together (Fig. 3a).

Similarly to SF-1 protein results, the cytosolic protein content of P_450_Arom was strongly high in epithelial cells from endometriosis endometria in basal condition, and also resistant to E_2_ and PGE_2_ presences (Fig. 3b). In control epithelial cells, P_450_Arom protein content was significantly increased by E_2_ (292 %), effect partially blocked by ICI pre-treatment. PGE_2_ increased (258 %) the protein content of P_450_Arom and the presence of both, E_2_ and PGE_2_, shows an additive effect (525 %) on the protein content (Fig. 3b).

### Specific agonists of estrogen receptors involved on USF2, SF-1 and P_450_Arom protein content by E_2_ stimulation

Taking into account that cells obtained from endometriosis women are highly endogenously stimulated, control epithelial cells were used for the following experiments to assess the ER isoform involvement. For that, control cells were incubated with specific agonists for ERα (PPT), ERβ (DPN) and GPER1 (G1).

**Fig. 4:**
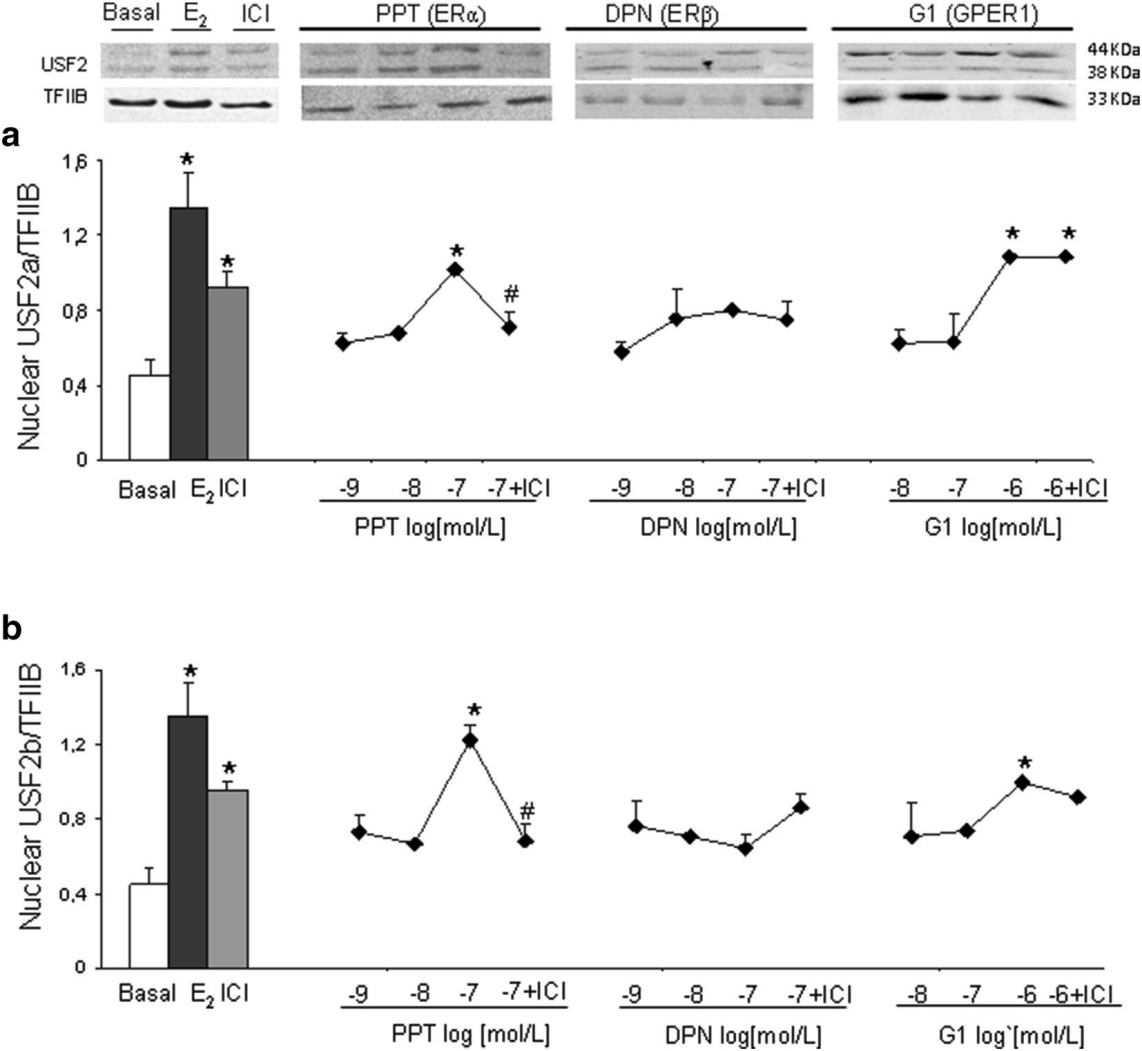
Dose-response curves of specific agonists on USF2a (**a**) and USF2b (**b**) nuclear protein content. Endometrial epithelial cells obtained from 4 control women were treated for 24 h with E_2_ (10^−8^ mol/L), PPT (10^−9^ to 10^−7^ mol/L), DPN (10^−9^ to 10^−7^ mol/L), and G1 (10^−8^ to 10^−6^ mol/L) in the presence or absence of ICI (10^−6^ mol/L; 40 min previously added). Representative immunoblot is shown. Data were normalized with TFIIB. Results are the mean ± SEM of EEC obtained from at least 4 control women. *p < 0.05 vs. basal; ^#^p < 0.05 vs. agonist

These cells responded to PPT at 10^−7^ mol/L and G1 at 10^−6^ mol/L, increasing the protein content of USF2a by 153 and 164 % and USF2b by 169 and 109 %, respectively (p < 0.05). The stimulatory effect of PPT was blocked by the presence of ICI. Paradoxically, ICI alone increased both USF2 variants. No significant effect was observed with DPN (Fig. 4a, b).

**Fig. 5:**
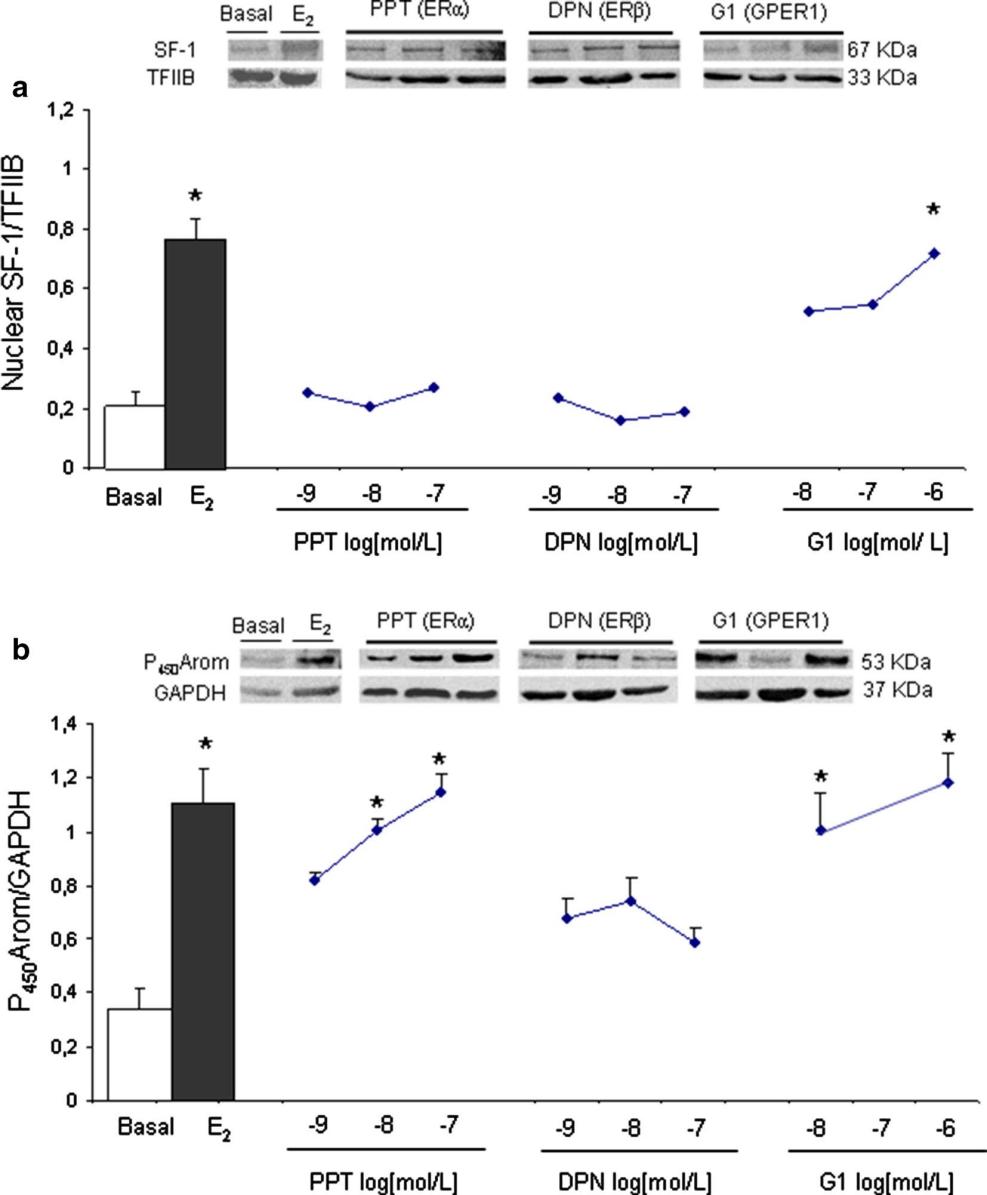
Dose-response curves of specific agonists on SF-1 (**a**) and P_450_Arom (**b**) protein content. Endometrial epithelial cells obtained from 4 control women were treated with E_2_ (10^−8^ mol/L), PPT (10^−9^ to 10^−7^ mol/L), DPN (10^−9^ to 10^−7^ mol/L), and G1 (10^−8^ to 10^−6^ mol/L). Representative immunoblots are shown. Data for SF-1 (nuclear homogenates) were normalized with TFIIB and for P450Arom (cytosol homogenates) with GAPDH. Results are the mean ± SEM. *p < 0.05 vs. basal

Only G1 increased SF-1 nuclear protein content by 250 % at 10^−6^ mol/L in the control cells (Fig. 5a). Similarly to SF-1, P_450_Arom cytosolic protein content was increased in a dose-dependent manner by G1 (242 %) and also by PPT (232 %) as compared to basal condition (Fig. 5b).

## Discussion

To our knowledge, this is the first report on human endometrial USF2a and USF2b protein co-expression throughout the menstrual cycle, and their positive regulation by E_2_ through ERα and GPER1.

The reduced nuclear protein content of USF2 variants during the late secretory phase in control endometria is consistent with the decreased plasma E_2_ and progesterone levels during this period of the menstrual cycle. In contrast, eutopic endometria from endometriosis patients exhibited high USF2 variants protein contents during this stage coincidently with the estrogenic microenvironment described in the eutopic and ectopic endometria of these patients [10, 13, 14, 30–32]. On the other hand, the opposite expression of USF2a and USF2b found in control endometria during the mid secretory phase, when the embryo implantation occurs, was not observed in eutopic endometria from endometriosis women, which may contribute to the infertility associated to this pathology. The different expression patterns of endometrial USF2 variants between women with and without endometriosis throughout the menstrual cycle add new molecules to those abnormally expressed in this tissue as has been widely reported [33–36]

We observed a strong E_2_-stimulatory effect on USF2 variants nuclear protein contents in epithelial cells from control endometria. These findings are supported by the epithelial cells response to ICI, an antagonist of ERα and ERβ, which partially blocked those effects induced by E_2_, but completely blocked those induced by PPT (specific agonist of ERα) and acting as agonist for GPER1, unaffected those effects induced by G1 (specific agonist of GPER1), confirming the dual action of ICI on estrogen receptors [37]. These data suggest that this process is under ovarian steroid regulation through the classic nuclear ERα and also GPER1. Interestingly, positive regulation of the ERα expression by USF2 has been reported in sheep uterine arteries [32, 38] showing a complex relationship between both transcription factors. The action of E_2_ through GPER1 not only may be involved on the USF2 protein synthesis or viability, but also on the USF2 activation through several pathways described for GPER1 [25, 39–41]. This aspect is of high relevance considering the important role, beside the cell-specificity, that specific phosphorylation plays on the activation of USF protein that modifies its function from tumor suppressor in prostate cancer to tumor promoter in lung cancer and thyroid cancer as recently Horbach et al. reported [15, 42].

Our first hypothesis was that the strong increase of USF2 induced by E_2_ might stimulate the SF-1 transcription activating the E-box motif, which in turn may induce Cyp19A1 gene increasing the key enzyme P_450_Arom. However, the discordance between the high USF2 protein expression and the weak binding observed on SF-1 E-Box DNA consensus studies suggests a partial effect of USF2 on SF-1 gene promoter in cell treated with E_2_. Nevertheless, more studies are needed to confirm or to discard this pathway. Furthermore, the proinflammatory environment, generated by PGE_2_, was unable to modify USF2 variants protein, although induced a strong stimulation on SF-1 and P_450_Arom protein contents, indicating different regulations.

The up-regulation of P_450_Arom by PGE_2_ through cAMP/CREB signaling pathway was previously reported [1, 11, 12, 43]. The additive effect of E_2_ and PGE_2_ on P_450_Arom protein contents indicates different activation mechanisms. Similar additive effect we reported previously in isolated control epithelial cells treated with peritoneal fluid from endometriosis women (PF-E) and Bu_2_cAMP [44] mimicking the conditions of the endometriotic lesions. In our control epithelial cell model, P_450_Arom stimulation by E_2_ was through ERα and GPER1, but not through ERβ as it was previously proposed [1, 45] probably by the use of isolated control epithelial cells and not endometriotic stromal cells. It is known the important role of SF-1 on steroid hormone biosynthesis, and also on development, differentiation, and function of the endocrine tissues [46]. The non-classic receptor GPER1 mediating the E_2_ stimulatory action on SF-1 protein content as shown by our G1 data, is in agreement with SF-1 activation and endometrial cell proliferation through the PI3K and MAPK pathways activated in several cell lines transfected with GPER1 [39, 40]. However, cAMP pathway cannot be ruled out according to similar response to (Bu)_2_cAMP of control or SF-1-transfected endometrial epithelial cells as we previously reported [44].

In the present study, control epithelial cells were sensitive to E_2_ and/or PGE_2_ treatments, mimicking the estrogenic and pro-inflammatory microenvironment described in endometriosis, inducing abnormal molecule expression similarly to endometria from women with endometriosis as has been widely reported by several authors including our own group [14, 33–36, 47]. Very little information are regarding USF2, and even less about USF2 variants. Our data of sustained USF2 protein expression during the secretory phase in eutopic endometria of women with endometriosis, an invasive estrogen-dependent disease, and the fact that the USF2 action is cell specific and may change its function from tumor suppressor to tumor promoter with invasive characteristics [15, 42], suggest that USF2 may be involved in the pathophysiology of the endometriosis.

## Conclusions

To our knowledge, this is the first report that shows USF2 variants protein expression patterns in human normal and pathologic endometria during the menstrual cycle and its E_2_ stimulation mediated by ERα and GPER1 visualized by the response of cells obtained from control endometria, being unaffected the endogenously stimulated cells from endometriosis origin. The lack of E_2_ stimulation on USF2/SF-1 E-Box/DNA-sequence binding and the absence of PGE_2_ effect on USF2 variants opposite to the strong induction that they exert on SF1 and P450 proteins suggest different mechanisms and regulations. The sustained USF2 protein expression during the secretory phase in eutopic endometria of women with endometriosis may participate in the pathophysiology of this disease strongly associated with infertility and its characteristic endometrial invasion to ectopic sites in the pelvic cavity.

## Methods

### Subjects

Eutopic endometrium was obtained from 37 women undergoing diagnostic laparoscopy for endometriosis associated with pain and/or infertility (endometriosis group), and 49 women without endometriosis undergoing laparoscopy for tubal ligation or hysterectomy for a benign non-endometrial gynecologic condition (control group) in the Clinical Hospital San Borja-Arriarán. The age of these women was 33.9 ± 5.6 years for the endometriosis group and 36.7 ± 6.5 years for the control group (p < 0.05). Both groups of women had abstained from any hormonal treatment for at least 3 months prior to surgery. Endometrial biopsies were obtained during surgery with Cornier pipelle suction curettage from the corpus of the uterus, kept in cold sterile phosphate buffer saline (PBS), and transported to the laboratory at 4 °C. One piece of the tissue was fixed in formalin for histological evaluation, others pieces were frozen for protein studies or used for endometrial epithelial cells isolation.

The endometriosis grade was 49 % minimal-mild (score 1–15 points) and 51 % moderate-severe (score ≥16 points) according to American Society of Reproductive Medicine criteria [48]. Endometriosis was diagnosed during surgery by visual evaluation by an experimented surgeon in each patient. This study was approved by the ethical committees of Faculty of Medicine of University of Chile and Metropolitan Central Health Service of Chile; each patient signed a written informed consent before surgery.

Endometrial samples were dated according to Noyes criteria [49] and classified as proliferative (days 6–14; 12 control and 9 endometriosis samples) phase or early (days 15–18; 12 control and 10 endometriosis), mid (days 19–23; 12 control and 10 endometriosis), and late secretory phase (days 24–28; 13 control and 8 endometriosis).

### Cell culture

Secretory endometrium was washed in PBS, minced, and digested according to previous indication [50]. The glands were separated and cultured according to previous indication [44, 50] and after the first or second passage, the cells were reseeded in duplicate protein studies until sub-confluence. Then, the cells were incubated in fetal bovine serum-free medium (defined-medium) for 24 h, and treated for another 24 h in fresh defined-medium without (basal) or with prostaglandin E_2_ (PGE_2_, 10^−8^ mol/L; Sigma), or E_2_ (10^−8^ mol/L; Sigma). Increasing concentrations of Propylpyrazole-triol (PPT, 10^−9^ to 10^−7^ mol/L, Tocris Bioscience, Bristol, UK) and Diarylpropionitrile (DPN, 10^−9^ to 10^−7^ mol/L, Tocris), specific agonists of ERα and ERβ, respectively, or G1 (10^−8^ to 10^−6^ mol/L, Merck KGaA, Darmstadt, Germany) specific agonist of GPER1, were also added to cell cultures for 24 h in the presence or absence of ICI-182,780 (10^−6^ mol/L, ERα and ERβ antagonist; Tocris) added 40 min before of ER agonists.

### Protein homogenate preparation

Cytosolic and nuclear proteins from endometrial pieces and epithelial cells were obtained as previously reported [47]. The protein concentration was determined using the Bradford Assay reagent (BioRad, Hercules, CA, USA).

Thirty μg of cytosolic and nuclear proteins were denatured, resolved in 10 % PAGE-SDS, and electrotransferred into nitrocellulose membranes (BioRad) as previously indicated [44, 50]. After blocking with 5 % BSA, the membranes were incubated overnight at 4 °C with primary antibodies against USF2 (polyclonal, 1:800; Abcam Inc, Cambridge, MA, USA), SF-1 (polyclonal, 1:800; ABR Affinity BioReagents, Golden, CO., USA), P_450_Arom (monoclonal; 1:600; Serotec, Oxford, UK), TFIIB (monoclonal, 1:500; BD Biosciences, MD, USA), or GAPDH (polyclonal; 1:5000; Abcam). The images were captured with Discovery10gD (Ultralum, Claremont, CA, USA) using UltraQuant 6.0.0.344 software, analyzed with CarestreamMI5.0.6.20 software (Carestream Health, Inc., Rochester, NY, USA). The results were normalized with GAPDH or TFIIB analysis for cytosolic or nuclear extracts, respectively.

### SF-1 E-Box DNA shift assay

The assay was performed using LightShift Chemiluminescent EMSA kit (Thermo Scientific, Rockford, IL, USA). Briefly, 5 µg nuclear proteins obtained as described above were incubated during 20 min at room temperature in a reaction mix which included 20 fmol biotin end-labeled oligonucleotides that represented the SF-1 gene promoter containing the E-box (Integrated DNA Technologies, Inc., Coralville, IO, USA) following the manufacturer’s indications and as described Utsunomiya et al. [17] For supershift study, nuclear proteins were previously incubated with 1 µg USF2 antibody (Abcam) during 2 h at 4 °C. The samples were resolved in non denaturing 4 % polyacrylamide gel, electrotransferred to biodyne-B membrane (Pall Corporation, Port Washington, NY, USA), which was UV-light crosslinked (UVP HL-2000 HybriLinker, Cambridge, UK), blocked and the label detected following the manufacturer’s indications (Thermos). The images were captured with Discovery10gD using UltraQuant 6.0.0.344 software.

### Statistical analysis

Results are expressed as mean ± SEM. Kolmogorov–Smirnov test was used to evaluate normal distribution. When non-parametric distribution was present Mann–Whitney or Kruskal–Wallis tests were used, followed by a Dunn test. Means were expressed as percent of increase. Analysis of covariance (ANCOVA) was employed to test statistical interaction with co-variables like age and phases of the menstrual cycle.

## Abbreviations

USF2: upstream stimulatory factor
SF-1: steroid factor-1
GPER1: G-coupled protein estrogen receptor 1
P_450_Arom: aromatase enzyme
PPT: 4,4′4″-(4-Propyl-[1*H*]-pyrazole-1,3,5-triyl)*tris*phenol
DPN: 2.3-bis(4-Hydroxyphenyl)-propionitrile
ICI 182,780: 7α,17β-[9[4,4,5,5,5-Pentafluoropentyl]sulfinyl]nonyl]estra-1,3,5(10)-triene-3,17-diol
G1: 1-(4-(6-Bromobenzo[1,3]dioxol-5-yl)-3a,4,5,9b-tetrahydro-3H-cyclopenta[c]quinolin-8-yl)-ethanone
E_2_: estradiol
PGE_2_: prostaglandin E_2_
ER: estrogen receptor
